# Social encounters produce different autonomic response between dominants and subordinates in crows

**DOI:** 10.1098/rsos.220972

**Published:** 2022-10-19

**Authors:** Kazuaki Takeda, Nana Takahashi, Ei-Ichi Izawa

**Affiliations:** ^1^ Department of Psychology, Keio University, Tokyo 108-8345, Japan; ^2^ Japan Society of the Promotion for Science, Tokyo, Japan

**Keywords:** autonomic nervous system, birds, dominance, heart rate variability, vagal tone, electrocardiogram

## Abstract

Recent studies of behavioural physiology on animals have suggested the crucial role of peripherally physiological signals in eliciting arousal and emotion. Heart rate (HR) is one of the useful and critical signals to measure autonomic regulation as a physiological basis for arousal and emotion in response to biologically significant stimuli such as social encounter with conspecific individuals. However, our understanding of peripherally physiological response such as HRs and autonomic activities under social contexts of non-human animals is still limited, particularly in birds. Here, we examined the autonomic activity of behaving crows exposed to a dominant and a subordinate conspecific by using non-invasive electrocardiogram recording. We found different patterns of autonomic responses dependent on the relative dominance position: dominant crows encountering subordinates showed the elevation of sympathetic activity, whereas subordinates encountering dominants showed decreased HR with elevated parasympathetic activity. This is the first study in birds to report different autonomic responses dependent on relative dominance positions during dyadic social encounters. The present study advances our understanding of the role of the peripheral autonomic system, as an interactive system with the brain, in eliciting emotion/arousal associated with socially challenging environments from an evolutionary perspective.

## 1. Introduction

For humans and non-human animals, autonomic regulation, including heart rate (HR) changes, plays a crucial role in the homeostatic control of the physiological state of the body against external environmental challenges [1,2]. Autonomic functions in social behaviour have been suggested to play a critical role as a peripheral physiological signal for animals to regulate arousal level and to generate positive/negative emotion associated with encountered conspecifics. Both are involved in cognitive processes to produce appropriate behavioural reaction such as fight-or-flight and freezing behaviour [2–4].

To understand the evolution of the physiological basis for emotion and social behaviour, a growing body of research on non-human animals has examined HR responses during encountering or interacting with conspecific individuals, such as agonistic interactions between rats (*Rattus norvegicus*) [5], domestic pigs (*Sus scrofa domesticus*) [6], macaques (*Macaca mulatta*) [6], domestic chicken (*Gallus domesticus*) [7], greylag geese (*Anser anser*) [8,9] and king penguins (*Aptenodytes patagonicus*) [10]. These previous studies reported that HR increased during encountering and interacting conspecifics in agonistic encounters. These findings suggest that autonomic response with the higher activity of sympathetic nervous system (SNS) and lower activity of parasympathetic nervous system (PNS) occurs toward socially stressful events and stimuli.

However, the decrease of HR, which is regulated to balance lower sympathetic activity and higher vagal tone during social encounter and interaction, has been reported in two different lines of social behaviour or situation. One is socially tension-reduced, affiliative behaviour such as social grooming in horses (*Equus caballus*) [11], cows (*Bos taurus*) [12] and macaques (*M. nemestrina*, *M. mulatta*) [13,14]. HR decrease in these instances is thought to be associated with socially relaxed, less tensioned relationships or contexts between conspecifics. On the other hand, HR decrease has also been known to occur in association with a defensive state, typically shown as orienting response or freezing behaviour, towards the expectation or presence of threatening or aversive stimulus [4]. These instances include encountering a predator in wild rodents [15] and in incubating hens [16], conditioned fear in rats [17] and mice [18], and brain stimulation-induced freezing in rats [19]. To our knowledge, however, there has been no previous study that reported HR decrease in socially stressful situations such as agonistic encounters between dominants and subordinates. In such socially agonistic situations, HR decrease in subordinates could be expected because encountering dominant conspecifics should be aversive for subordinates, while HR in dominants could not be reduced since subordinates should not be aversive for dominants. However, such comparative analysis of autonomic response between dominants and subordinates during agonistic encounters was not reported particularly in birds.

To investigate the difference in autonomic response depending on relative dominance position, crows are ideal birds because they form complex social structures based on stable dominance rank and affiliative relationships between individuals [20–22]. In this study, we tested whether dominants and subordinates showed different patterns of autonomic response during agonistic encounters in large-billed crows (*Corvus macrorhynchos*). Previous studies using a dyadic encounter experiment revealed that males of captive large-billed crows formed clear dominance relationships in the first few encounters [23] and, once formed, maintained them for years [21]. It was also found that in captive groups of large-billed crows, dominance relationships among males were structured into a linear form and dominance rank was correlated with faecal corticosterone levels [24], suggesting the different activity states of hypothalamic–pituitary–adrenal axis (HPA axis) between males with different dominance ranks. Different levels of neuronal activities quantified with an immunohistochemical technique between dominants and subordinates of large-billed crows were found after dyadic exposure to each other in several nuclei of social brain networks which have been known to be involved in various social behaviours in vertebrates [25,26].

These previous findings of different activity of brain and endocrine systems between dominants and subordinates allow us to predict different patterns of autonomic activity during social encounters between dominant and subordinate males of large-billed crows. To evaluate autonomic activity based on HR data, we used time-domain parameters of HR variability (HRV) analysis such as standard deviation of the inter-heartbeat interval of normal sinus beats (SDNN, ms) as a proxy of autonomic activity, root mean square of the successive differences (RMSSD, ms) as a proxy of parasympathetic activity, and the ratio of SDNN to RMSSD (SDNN/RMSSD) as a proxy of sympathetic activity [27]. Using HR and HRV parameters, we predicted that socially dyadic encounters would cause (i) dominants to show higher SNS and lower PNS activity, which was supported by an increase of HR, SDNN and SDNN/RMSSD, as reflected by their aggressive state and (ii) subordinates to exhibit lower SNS and higher RMSSD activity, which was supported by a decrease in HR and increase in SDNN and RMSSD, as reflected by their defensive state. To test these predictions, we investigated autonomic activity of dominant and subordinate males of large-billed crows in a dyadic social encounter experiment using a non-invasive electrocardiogram (ECG) recording system.

## 2. Methods

### 2.1. Animals and housing

The study included 12 adult male large-billed crows (3–4 years old, 640–800 g body weight; electronic supplementary material, table S1). The sex of the birds was determined by DNA from blood samples. All of these birds were captured at the first yearlings during October 2015–February 2016 and October 2016–January 2017 in Tokyo and neighbouring areas with authorization from the Japanese Ministry of the Environment (No. 30030019, 03114001). After being captured, the birds were group-housed in one of four outdoor aviaries (5 m × 10 m × H 3 m) with other crows as a 5–10 bird group for other studies (electronic supplementary material, table S1). Three–six months prior to this experiment, crows were transported to individual cages (W 43 cm × D 60 cm × H 50 cm) placed in an animal housing room, in order to prevent the formation of dominance relationships and/or affiliative relationships between the crows outside this experiment. To reduce the individual housing stress, the individual cages were placed side-by-side in the room, which were visible and audible to each other. In the cages, the crows were provided with water and food *ad libitum*; they were fed dry food, raw meat and vitamin supplements. The animal housing room was maintained at 21 ± 2°C in a 13L : 11D cycle, with a light onset at 0800. The experimental and housing protocols adhered to the Japanese National Regulations for Animal Welfare and were approved by the Animal Care and Use Committee of Keio University (No. 21019). After the present study, the crows were kept in individual cages for another study but were routinely provided the opportunity to interact with 2–3 crows in an outdoor aviary.

### 2.2. Determination of relative dyadic dominance

**Figure 1.**
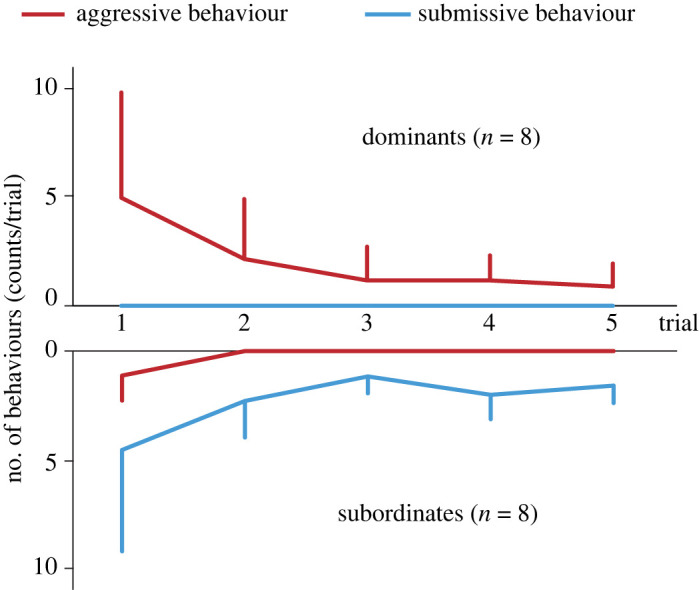
The numbers of aggressive (red) behaviours (jab, peck, aggressive vocalization and displacing approach) by the dominant (top) and submissive (blue) behaviours (submissive begging vocalization and avoidance) by the subordinates (bottom) symmetrically decreased through the five encounters. Top: dominants showed the decreasing trends of aggressive behaviour towards the subordinate (*χ*^2^ = 20.84, d.f. = 4, *p* < 0.001, non-parametric Friedman ANOVA) but no submission across five of the counters. Bottom: conversely, subordinates exhibited the decreasing trends of submissive behaviour to the dominants across the five encounters (*χ*^2^ = 21.17, d.f. = 4, *p* < 0.001) but were aggressive only on the first encounter.

Prior to the ECG recording sessions, the relative dominance position of each crow within dyads (i.e. a dominant and subordinate in each pair) was determined through three trials of a 5 min social encounter in an experimental room (W1.6 × D1.5 × H2.0 m^3^) in a similar procedure to our previous study [23,26]. Twelve dyads composed of 12 individuals were undertaken for this social encounter experiment. We chose the 12 dyads, each of which had no history of direct social interactions with each other in other past experiments and/or cohabitation in group-housing prior to this study (table 1; electronic supplementary material, table S1). This aimed to examine the physiological response primarily dependent on the dominance position but removing the other factors such as different tension levels due to different time length after dominance formation and a tension-reducing effect by an affiliative relationship established via cohabitation. Each encounter trial was conducted between 1000 and 1300. We introduced 2- to 4-day intervals between the successive encounters of each dyad. For each encounter trial, a winner and a loser were determined according to the criterion which was used in the previous studies [23,26]. Specifically, the loser was identified as the individual to exhibit submissive behaviours (i.e. submissive begging vocalization and avoidance) in response to the aggressive behaviour (i.e. jab, peck, aggressive vocalization and displacing approach) or even no explicit aggression by either individual, and then the other individual was considered as the winner. Within a dyad, an individual who won all of the initial three trials was defined as dominant, and the other one was defined as subordinate. These social behaviours during each trial were recorded by video cameras viewed from the side and top for offline behavioural coding by either of the experimenters (K.T., N.T. or E.I.). A dyad in which either individual won twice or fewer times with including ties was treated as an unclear dominance relationship and excluded from ECG recording. Out of the 12 dyads, eight showed the clear asymmetry of win/loss numbers in the three trials (i.e. 3 versus 0) and were considered as the formation of dominance relationship (figure 1 and the result for details). The other four dyads did not result in such asymmetric win/loss outcomes and were treated as unclear dominance. Therefore, the eight dyads, comprising 12 birds, with the clear dominant relationship were undertaken for the ECG recording in the subsequent fourth and fifth trials (table 1). Eight of the 12 birds were tested only once for ECG recording as either a dominant or a subordinate in one dyad, whereas the other four birds (A, D, E and Y) were tested twice in two dyads: birds Y and E, and bird D as dominants and subordinates, respectively, while bird A participated as a dominant in one dyad (A versus S) but as a subordinate in another (Y versus A).

**Table 1.** Dyads for ECG recording. Letters indicate individual birds. Birds A, D, E and Y included in two different dyads.

dominant	subordinate
A	S
P	V
Y	J
Y	A
G	D
H	W
E	D
E	R

### 2.3. Electrocardiogram recording device

**Figure 2.**
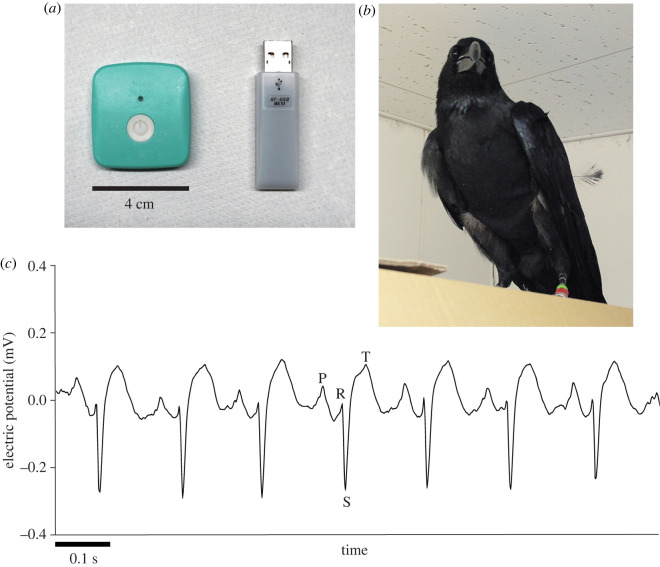
(*a*) Left ECG sensor/transmitter. Right: USB receiver. (*b*) A crow being fitted with a custom-made jacket. The small transmitter was attached to the chest skin of the crow. (*c*) ECG waves successfully recorded from a behaving crow. Letters (P, R, S, T) on the wave line indicate a series of peaks comprising single heartbeats. IBIs were defined as the temporal distance between adjacent S peaks.

ECG was recorded from one (i.e. the focal) of two socially interacting crows using a detachable sensor. A small wireless ECG sensor (RF-ECG2, GM3 Co., Japan; 4 × 3.5 × 0.7 cm^3^, 12 g, 512 Hz sampling frequency: figure 2*a*) with gel pad electrodes (Blue Sensor SP-00-S, Ambu, Denmark; ϕ 38 mm), which were trimmed suitably to a small size, was attached to the chest skin of the crows. The chest skin on the left side of the crows was exposed by shaving feathers in an area sized 4 × 4 cm^2^ under topical anaesthesia (Xylocaine Jelly 2%, AstraZeneca K.K., Japan) 2 days before the ECG recording experiment. The sensor was attached to the upper and caudal precordium of the crows. To prevent the sensor from being removed by foot scratching during ECG recording sessions, the crows were put in a custom-made jacket that covered the front trunk below the neck but allowed movement of the wings and feet (figure 2*b*). The crows were acclimated to the jackets in the experimental chamber three or more times prior to the experiment. ECG data were transmitted to a USB-type receiver connected to a PC and recorded in real time. Using this system, the ECG was recorded with Q-R-S components (RS type in lead II) [28], which enabled us to measure inter-beat intervals (IBI) based on S peaks (figure 2*c*).

### 2.4. Electrocardiogram recording session

Two to three days after the third encounter trials to determine the dominance relationship, two additional trials for each dyad were conducted for ECG recording with 2- to 3-day inter-trial intervals: one trial for the ECG recording from a dominant and the other from a subordinate. Each ECG recording trial was 13 min long. A few minutes before the start of each trial, the researcher gently attached an ECG sensor with a jacket on a crow. The session started with the introduction of the crow into the experimental room by the researcher. In each trial, the first 8 min of ECG recording was treated as *the pre-encounter phase*, during which an individual for ECG recording was placed alone. The next 5 min was treated as *an encounter phase*, where the other individual was introduced into the room immediately after the end of the pre-encounter phase, so that two birds could freely interact. After the end of the encounter phase, the birds were returned to their home cages without the ECG sensor and jacket. To exclude the handling effect of the ECG sensor attachment by the researcher on the physiological state of the crow, ECG data during the first 3 min of the pre-encounter phase were discarded from the analysis. This was because in a pilot experiment using other individuals not used in this study, where crows were placed alone for 6–8 min in the arena, we confirmed that it took 3 min after the crows entered to the arena for HR responses to reach the baseline level. Thus, we removed the first 3 min of the 8 min pre-counter phase from the analysis. All experiments were conducted between 1100 and 1400 h. The behaviour of the crows during the encounters was video-recorded (HDR-MV1, SONY, Japan) from the top of the room for offline coding. Behavioural coding for each trial was conducted by either of the three experimenters. The inter-coder reliability was *κ* = 0.95.

## 3. Analysis

### 3.1. Behaviour

During each encounter phase of the ECG recording trial, we counted the number of initiated and received aggressive behaviours and submissive behaviours of the ECG-recorded crows from the video data. To measure the non-social behavioural activity (such as walking) as a potential effective factor for HR and HRVs, we also calculated the total movement distance of the ECG-recorded crow separately during pre- and encounter phase based on the video-tracking data using the software UMATracker [29–31].

### 3.2. Heart rate variability

We performed HRV analysis using time-domain variables to examine autonomic activity. Specifically, we calculated the mean HR (beats min^−1^), s.d. of the IBI of normal sinus beats (SDNN, ms) as a proxy of autonomic activity, root mean square of the successive differences (RMSSD, ms) as a proxy of parasympathetic activity and the ratio of SDNN to RMSSD (SDNN/RMSSD) as a proxy of sympathetic activity, separately for 5 min before and during social encounters.

### 3.3. Statistics

To compare the autonomic activity during social interactions between dominant and subordinate individuals, we applied generalized linear mixed models (GLMMs) with Gamma error distribution with logit link function separately to the mean HR (beats min^−1^) and HRV indices such as SDNN, RMSSD and SDNN/RMSSD. Each model included one of the physiological indices (SDNN, RMSSD, SDNN/RMSSD and HR) as the response variable, *phase* (pre-interaction or encounter), *dominance* (dominant or subordinate), total movement distance, the *phase × dominance* interaction as the explanatory variables and *individuals* as a random factor. Social behaviour (aggression and submission) was not included as an explanatory variable in the models because those behaviours occurred only in a few trials of ECG recording (see 4th and 5th trials in figure 1) and were considered insufficient predictors of the autonomic activities. If the *phase × dominance* interaction was significant, we performed two separate GLMMs on the dominant and subordinate datasets to examine the significant effects on the HRVs of dominant and subordinate crows. The total movement distance was log-transformed to fit a normal distribution. The significance of variables in each model was evaluated based on the Wald *χ^2^* statistics. All statistical analyses were performed using R v.4.2.2 with the package ‘lme4’ [32].

## 4. Results

**Figure 3.**
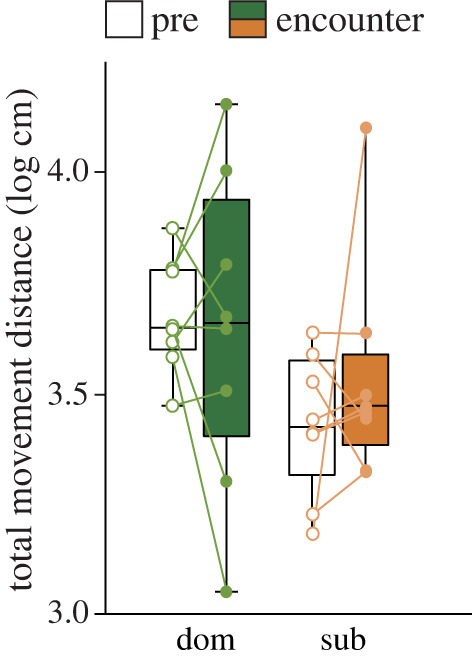
Total movement distance of ECG-recorded dominant (dom) and subordinate (sub) birds during pre (open) and encounter (coloured) phases. The upper, median and bottom of the boxes show the 75, 50 and 25 percentiles, respectively, with whiskers representing the maximum and minimum values.

Out of 12 dyads tested, eight dyads formed a clear dominance relationship in the initial three trials. Such clear dominance was characterized by the asymmetric patterns of the number of aggressive and submissive behaviours by the dominants and subordinates, respectively, across the trials (figure 1). We tested these eight dyads for ECG recording in the fourth and fifth trials. During the ECG recording trials, the total movement distance of ECG-recorded birds was not significant between the dominant and the subordinates and between pre- and encounter phases (figure 3). A linear mixed model, including log-transformed movement distance as a response variable, dominance, phase and their interaction as independents variable, and individuals as a random factor, produced no significant effect of any variables (*dominance* × *phase*, coefficient ± s.e. = −0.106 ± 0.181, *p* = 0.56, ns; *dominance*, coefficient ± s.e. = −0.141 ± 0.128, *p* = 0.28, ns; *phase*, coefficient ± s.e. = 0.002 ± 0.128, *p* = 0.97, ns).

**Figure 4.**
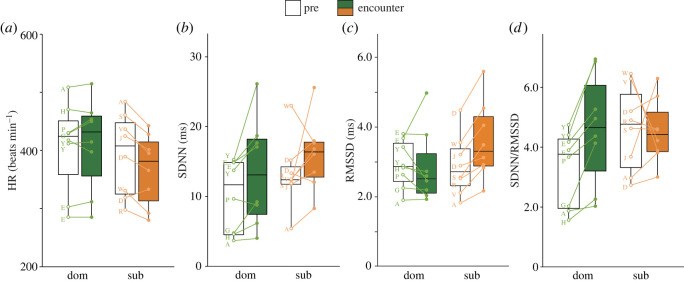
HR (*a*), SDNN (*b*), RMSSD (*c*) and SDNN/RMSSD (*d*) of dominants (dom) and subordinates (sub) before (open box) and during (filled box) encounters. Plots with lines on each box represent individual data. The upper, median and bottom of the boxes show the 75, 50 and 25 percentiles, respectively, with whiskers representing the maximum and minimum values. The letters on plots indicate individual birds (table 1).

GLMM analyses on HR and HRV variables revealed that dominants and subordinates showed the different response of HR, RMSSD and SDNN/RMSSD during the encounter. The GLMM analysis of the HR showed a significant interaction of *dominance × phase* (*p* = 0.046; table 2). Thus, we ran GLMMs separately on datasets of dominants and subordinates. The GLMM on dominants produced the significant effect of *movement distance* with a positive coefficient estimate (estimates ± s.e. = −0.084 ± 0.037, *p* = 0.023; table 3). The GLMM on subordinates revealed the significant effects of *phase* with a positive coefficient (estimates ± s.e. = 0.057 ± 0.027, *p* = 0.032; table 3 and figure 4*a*) and also *movement distance* with a negative coefficient (estimates ± s.e. = −0.487 ± 0.169, *p* = 0.004; table 3). These results suggest that HR of dominants was affected by how much the individuals moved during the trials and that the HR of subordinates decreased when encountering the dominants and by distance moved. Regarding SDNN, the GLMM yielded significant effects of both *dominance* with a positive coefficient (estimates ± s.e. = 0.563 ± 0.143, *p* < 0.001; table 4 and figure 4*b*) and *phase* with a negative coefficient (estimates ± s.e. = −0.274 ± 0.073, *p* < 0.001 l table 4 and figure 4*b*) but no significance of other variables. This suggests that SDNN of subordinates was higher than that of dominants and that both dominants and subordinates increased during encounters. The GLMM of RMSSD exhibited a significant interaction of *dominance × phase* (*p* < 0.001; table 5). Subsequent GLMM analyses separated on the datasets of dominants and subordinates revealed a significant effect of *phase* with a positive coefficient (estimates ± s.e. = 0.303 ± 0.115, *p* = 0.009; table 6 and figure 4*c*) and significant effects of *phase* and *movement distance* (*phase*, estimates ± s.e. = −0.204 ± 0.061, *p* < 0.001; *movement distance*, estimates ± s.e. = 0.837 ± 0.387, *p* = 0.031; table 6 and figure 4*c*), respectively. These results suggest that RMSSD of both dominants and subordinates correlated with their movement distance and that RMSSD of subordinates increased by the presence of dominants but that of dominants did not. The GLMM of SDNN/RMSSD showed a significant interaction of *dominance × phase* (*p* = 0.014, table 7). Separate GLMM analyses on dataset of dominants produced a significant effect of *phase* with a negative coefficient estimate (estimates ± s.e. = −0.332 ± 0.048, *p* < 0.001; table 8 and figure 4*d*) and, on datasets of subordinates, a significant effect of *movement distance* with a negative coefficient (estimates ± s.e. = −1.118 ± 0.465, *p* = 0.016; table 8). These results suggest that the SDNN/RMSSD of the dominants increased during encounters with subordinates whereas that of the subordinates did not, and that the SDNN/RMSSD of subordinates negatively correlated with *movement distance*.

**Table 2.** Outputs of GLMM for HR.

variables	estimates	s.e.	*t*-value	*p*
intercept	*1.437*	*0.247*	*5.810*	*< 0.001*
dominance^a^	*−0.144*	*0.054*	*−2.680*	*0.007*
phase^b^	−0.010	0.027	−0.365	0.715
movement distance	−0.004	0.066	−0.068	0.946
dominance × phase	*0.0761*	*0.038*	*1.991*	*0.046*

Italicized indicates statistically significant results.

^a^*Dominant* was set to 0.

^b^*Encounter* was set to 0.

**Table 3.** Outputs of separate GLMMs on *dominant* and *subordinate* dataset.

variables	estimates	s.e.	*t*-value	*p*
*dominants*				
intercept	*1.157*	*0.163*	*7.083*	*< 0.001*
phase^a^	−0.010	0.014	−0.769	0.442
movement distance	*0.084*	*0.037*	*2.274*	*0.023*
*subordinates*				
intercept	*2.959*	*0.588*	*5.034*	*< 0.001*
phase^a^	*0.057*	*0.027*	*2.139*	*0.032*
movement distance	*−0.487*	*0.169*	*−2.884*	*0.004*

Italicized indicates statistically significant results.

^a^*Encounter* was set to 0.

**Table 4.** Outputs of the GLMM for SDNN.

variables	estimates	s.e.	*t*-value	*p*
intercept	1.218	0.653	1.864	0.062
dominance^a^	*0.563*	*0.143*	*3.922*	*< 0.001*
phase^b^	*−0.274*	*0.073*	*−3.760*	*< 0.001*
movement distance	0.288	0.176	1.637	0.101
dominance × phase	0.067	0.103	0.651	0.515

Italicized indicates statistically significant results.

^a^*Dominant* was set to 0.

^b^*Encounter* was set to 0.

**Table 5.** Outputs of the GLMM for RMSSD.

variables	estimates	s.e.	*t*-value	*p*
intercept	−0.292	0.492	−0.594	0.553
dominance^a^	*0.234*	*0.110*	*2.134*	*0.033*
phase^b^	0.055	0.056	0.979	0.328
movement distance	*0.357*	*0.134*	*2.659*	*0.008*
dominance × phase	*−0.268*	*0.080*	*−3.360*	*< 0.001*

Italicized indicates statistically significant results.

^a^*Dominant* was set to 0.

^b^*Encounter* was set to 0.

**Table 6.** Outputs of separate GLMMs on dominant and subordinate dataset.

variables	estimates	s.e.	*t*-value	*p*
dominants				
intercept	−0.220	0.440	−0.499	0.618
phase^a^	0.056	0.044	1.261	0.207
movement distance	*0.303*	*0.115*	*2.623*	*0.009*
subordinates				
intercept	−1.679	1.336	−1.256	0.209
phase^a^	*−0.204*	*0.061*	*−3.311*	*< 0.001*
movement distance	*0.837*	*0.387*	*2.162*	*0.031*

Italicized indicates statistically significant results.

^a^*Encounter* was set to 0.

**Table 7.** Outputs of the GLMM for SDNN/RMSSD.

variables	estimates	s.e.	*t*-value	*p*
intercept	1.604	0.831	1.930	0.054
dominance^a^	0.301	0.169	1.776	0.076
phase^b^	*−0.331*	*0.094*	*−3.495*	*< 0.001*
movement distance	−0.089	0.226	−0.393	0.694
dominance × phase	*0.329*	*0.134*	*2.451*	*0.014*

Italicized indicates statistically significant results.

^a^*Dominant* was set to 0.

^b^*Encounter* was set to 0.

**Table 8.** Outputs of separate GLMMs on dominant and subordinate dataset.

variables	estimates	s.e.	*t*-value	*p*
dominants				
intercept	0.611	0.535	1.143	0.253
phase^a^	*−0.332*	*0.048*	*−6.908*	*< 0.001*
movement distance	0.175	0.132	1.325	0.185
subordinates				
intercept	*5.350*	*1.601*	*3.340*	*< 0.001*
phase^a^	0.0047	0.112	0.042	0.966
movement distance	*−1.118*	*0.465*	*−2.404*	*0.016*

Italicized indicates statistically significant results.

^a^*Encounter* was set to 0.

## 5. Discussion

The present study examined autonomic activity based on HR data during dyadic encounters between dominant and subordinate male large-billed crows. Autonomic activity included the time-domain parameters of HRV analysis, such as SDNN, RMSSD and SDNN/RMSSD as a proxy of autonomic activity, parasympathetic and sympathetic activity, respectively. We predicted that during dyadic encounters, autonomic activities of (i) the dominants would show higher SNS and lower PNS activity, accompanied by an increase in HR, SDNN and SDNN/RMSSD, as reflected by their aggressive state and (ii) the subordinates would exhibit lower SNS and higher RMSSD activity, accompanied by a decrease in HR and increase in SDNN and RMSSD, as reflected by their defensive state.

The present study provides the first evidence in birds of different patterns of autonomic responses during social encounters between dominants and subordinates. Dominants encountering subordinates showed no significant change in HR but an increase in both SDNN and SDNN/RMSSD, suggesting an elevation of SNS. By contrast, subordinates encountering dominants exhibited a decrease in HR and elevation of both SDNN and RMSSD, suggesting activation of both SNS and PNS. Additionally, these autonomic responses of both dominants and subordinates were correlated with *movement distance*, suggesting autonomic activation was accounted for not solely by psychological factors based on agonistic encounter but also by their own movement during encounters.

The present results of elevation in SDNN and SDNN/RMSSD in dominants during encountering subordinates suggest that the autonomic response in agonistic encounters to subordinates is regulated by higher SNS and lower PNS activity. This finding in dominants was consistent with previous studies reporting HR increases in agonistic encounters between conspecifics, given the physiological relevance of HR increases with higher SNS activity in general, although HRV parameters were not analysed in those studies [3,5–10]. The increased SNS activity in dominants during encountering subordinates may be affected partially by physical factors such as a higher level of behavioural activity because HR was positively correlated with movement distance. However, given that other HRV parameters than RMSSD were not affected by movement distance and that the dominants showed only a few numbers of aggressive behaviours toward subordinates in the two encounter trials, SNS elevation of the dominants during encountering the subordinates is likely to reflect psychophysiological responses to agonistic encounter situations.

Decreased HR with increased SDNN and RMSSD in subordinates during encounters suggest that autonomic responses in agonistic encounters involve higher PNS and lower SNS activity. This activity pattern of decreased HR and higher vagal tone was consistent with the autonomic response of animals in defensive states [4]. The similar autonomic response pattern was found in hens showing orienting/freezing responses to human approach [16], in mice receiving foot-shock stimuli under a fear conditioning paradigm [18] and in rats freezing generated by an electrical brain stimulation [19]. Given autonomic response associated with a defensive state supported by these previous findings, decreased HR with higher vagal tone found in subordinate crows during agonistic encounters in the present study could be associated with a defensive state, like freezing, of the subordinates by encountering socially aversive dominants.

HR decrease with elevated vagal tone has been suggested to be associated not only with defensive state but also with a tension-reduced state such as social affiliation [11–14]. However, HR decreases with increased PNS activity of subordinate crows in the present study cannot be explained by a tension-reduction effect because no affiliative behaviour was observed during the encounter and affiliative relationships between the individuals could not be formed outside the experiment due to individual housing conditions. Another possibility of HR reduction and higher vagal tone might be caused by a recovery effect of the subordinates to settle down to the experimental arena after being introduced there. However, this possibility is unlikely since our analysis excluded the initial 3 min of each 13 min trial, which HR of individuals alone in the arena took for a recovery to baseline levels and included the subsequent 10 min data of pre- and encounter phases. Such short-term recovery effects of HR, thus, could not account for lower HR and higher vagal tone in an encounter phase than those in a pre-encounter phase.

Autonomic responses such as a decrease in HR with higher vagal tone in subordinates during the encounter might be accounted for by movement. The results that HR and SDNN/RMSSD were correlated negatively to movement distance, while RMSSD was positively correlated suggest a potential relationship between more movement and higher PNS (and lower SNS). This potential relationship between movement and PNS in subordinates might reflect those subordinates in a defensive state moved sensitively as a reaction to the dominant's movement. In other words, the correlation between movement and autonomic response in subordinates might result not from physical factors, but from psychological factors associated with the defensive state of subordinates against dominants. The shorter movement distance during the encounter phase seen in figure 1 may reflect the postulated defensive/freezing state of subordinates encountering dominants. To test the potential relationship between movement and autonomic response in subordinates, causal structure of movement between dominants and subordinates in a shorter time scale (i.e. seconds) should be analysed in the future research.

The HRs of large-billed crows recorded in this study ranged from 280 to 510 beats min^−1^ during both pre-encounter and encounter phases. This range was slightly higher than those reported in other corvids, such as American crows (*C. brachyrhynchos*; 250–330 beats min^−1^) [33], fish crows (*C. ossifragus*; 300–400 beats min^−1^) [33], rooks (*C. frugilegus*; 300–390 beats min^−1^) [34] and carrion crows (*C. corone*; 220–480 beats min^−1^) [35], although individual and/or species variations may exist. In those previous studies, HRs were recorded from birds caught in the wild and temporarily encaged individually, under resting conditions (i.e. no explicit stimulus given). One possibility to account for the higher HRs in our study may be captive stress due to month-long individual housing. In general, corvids are neophobic [36] and stress from month-long individual housing may result in elevated HRs, even at the baseline level. Such stress-induced high baseline HRs in the pre-encounter phase may cause a ceiling effect, preventing further increase in HRs.

During the fourth and fifth encounters for ECG recording, dominants and subordinates ceased to show aggressive and submissive behaviours (figure 1). However, this lack of explicit behaviour did not necessarily indicate that the dominance relationships had collapsed, because aggressive and submissive behaviours, although few in number, occurred in a dominance position-dependent manner in the fourth and fifth trials. Similar behavioural trends among the repeated encounters were confirmed in a two- or three-month-long experiment using the dyadic encounter paradigm of large-billed crows [23]. Under group-housing conditions for 1 year or more, aggressive interactions between individuals became less frequent once a dominance relationship was formed (e.g. three times or less per dyad/60 min), but occurred consistently in a dominance-dependent manner, indicating that the dominance was stable even with a very low frequency of aggressive and submissive behaviours [21,24]. Given the stable dominance relationship confirmed in previous studies, the dominance relationships were maintained even with very few aggressive and submissive behaviours in the fourth and fifth encounters.

Although our findings of autonomic response were consistent with the previous reports, careful discussion should be necessary. This is because the present results contained the data from the same individuals repeatedly tested in two dyads (i.e. birds A, D, E and Y). Such repeated measurement might have facilitated the present findings of dominant- and subordinate-specific patterns of autonomic response. However, the individual data shown in figure 4 suggests that this may not be the case. Birds E and Y, which severed as dominants in two dyads, showed similar patterns in terms of significant HRV parameters, such as increased SDNN and SDNN/RMSSD compared with the different subordinate opponents. By contrast, bird D served as the subordinate in two dyads and exhibited a similar increase in RMSSD but exhibited different HR responses between the dyads. Interestingly, bird A, which served as a dominant in one dyad but as a subordinate in another, showed a clear autonomic response (i.e. HR decrease and RMMD increase) when acting as a subordinate, but showed no such response when acting as a dominant. These individual data, although not based on statistical analyses, suggest that the autonomic response of a particular individual may vary in response to a given opponent or relative dominance position.

However, given the individual difference in physiological stress-coping mechanisms including ANS, HPA axis and immune system in various animals [37–39], there is still a possibility that the present results might be facilitated by specific individuals such as with robust autonomic response. A possible future direction is, using a larger sample size, to investigate autonomic response-dependent dominance positions and their individual differences. Individual characteristics of autonomic response as a stress-coping mechanism might contribute to each individual to choose what social relationship could be formed with whom in a group. Integrative research on peripheral physiological mechanisms, such as ANS as in our study, together with brain mechanisms for stress coping in social environments in non-human animals could advance our understanding of the evolution of ‘emotion’ based on psychophysiological homeostatic systems.

## Data Availability

Data can be accessed at the following figshare site: https://doi.org/10.6084/m9.figshare.20372625.v1 [40]. Supplementary material is available online [41].
